# Analysis of the transcriptional activity of endogenous NFAT5 in primary cells using transgenic NFAT-luciferase reporter mice

**DOI:** 10.1186/1471-2199-9-13

**Published:** 2008-01-25

**Authors:** Beatriz Morancho, Jordi Minguillón, Jeffery D Molkentin, Cristina López-Rodríguez, Jose Aramburu

**Affiliations:** 1 Immunology Unit, Department of Experimental and Health Sciences, Universitat Pompeu Fabra, and Barcelona Biomedical Research Park-PRBB. Carrer Dr Aiguader 88, 08003 Barcelona, Spain; 2 Division of Molecular Cardiovascular Biology, Department of Pediatrics, Children's Hospital Medical Center. 3333 Burnet Ave, Cincinnati, OH 45229-3039, USA

## Abstract

**Background:**

The transcription factor NFAT5/TonEBP regulates the response of mammalian cells to hypertonicity. However, little is known about the physiopathologic tonicity thresholds that trigger its transcriptional activity in primary cells. Wilkins et al. recently developed a transgenic mouse carrying a luciferase reporter (9xNFAT-Luc) driven by a cluster of NFAT sites, that was activated by calcineurin-dependent NFATc proteins. Since the NFAT site of this reporter was very similar to an optimal NFAT5 site, we tested whether this reporter could detect the activation of NFAT5 in transgenic cells.

**Results:**

The 9xNFAT-Luc reporter was activated by hypertonicity in an NFAT5-dependent manner in different types of non-transformed transgenic cells: lymphocytes, macrophages and fibroblasts. Activation of this reporter by the phorbol ester PMA plus ionomycin was independent of NFAT5 and mediated by NFATc proteins. Transcriptional activation of NFAT5 in T lymphocytes was detected at hypertonic conditions of 360–380 mOsm/kg (isotonic conditions being 300 mOsm/kg) and strongly induced at 400 mOsm/kg. Such levels have been recorded in plasma in patients with osmoregulatory disorders and in mice deficient in aquaporins and vasopressin receptor. The hypertonicity threshold required to activate NFAT5 was higher in bone marrow-derived macrophages (430 mOsm/kg) and embryonic fibroblasts (480 mOsm/kg). Activation of the 9xNFAT-Luc reporter by hypertonicity in lymphocytes was insensitive to the ERK inhibitor PD98059, partially inhibited by the PI3-kinase inhibitor wortmannin (0.5 μM) and the PKA inhibitor H89, and substantially downregulated by p38 inhibitors (SB203580 and SB202190) and by inhibition of PI3-kinase-related kinases with 25 μM LY294002. Sensitivity of the reporter to FK506 varied among cell types and was greater in primary T cells than in fibroblasts and macrophages.

**Conclusion:**

Our results indicate that NFAT5 is a sensitive responder to pathologic increases in extracellular tonicity in T lymphocytes. Activation of NFAT5 by hypertonicity in lymphocytes was mediated by a combination of signaling pathways that differed from those required in other cell types. We propose that the 9xNFAT-Luc transgenic mouse model might be useful to study the physiopathological regulation of both NFAT5 and NFATc factors in primary cells.

## Background

NFAT5/TonEBP belongs to the Rel family of transcription factors, which also comprises NF-κB and the calcineurin-dependent NFATc proteins (NFAT1/NFATc2, NFAT2/NFATc1, NFAT3/NFATc4, NFAT4/NFATc3) [1,2]. Rel proteins have in common a conserved DNA binding domain, but do not display recognizable similarities outside of it. The DNA binding domain of NFAT5 is considered a hybrid between that of NF-κB and NFATc proteins, since it is a constitutive dimer, structurally similar to NF-κB, but has NFATc-like DNA sequence specificity, with its optimal binding site being a 5'-TGGAAA(C/A/T)A(T/A)-3' motif, in which the NFATc cognate element is 5'-(T/A/C)GGAA(A/G)-3' [2-4]. NFATc and NFAT5 differ substantially in their mechanisms of activation and biological function. NFATc proteins are characteristically activated by the phosphatase calcineurin in response to increases in intracellular calcium concentration [5,6], whereas NFAT5 is activated by hypertonicity [1]. Activation of NFAT5 is regulated by different kinases, such as the stress-activated kinase p38, Fyn [7], PKA [8], ERK [9], the PI3-kinase-related kinase (PIKK) ATM [10,11], and phosphoinositide 3-kinase (PI3-kinase) [11]. p38 has been shown to regulate NFAT5 in some cell types but not in others [7,12]. NFATc proteins play fundamental roles in the immune, nervous and cardiovascular systems (reviewed in [13-15]). NFAT5 allows mammalian cells to adapt to hypertonicity [16,17], by inducing the expression of osmoprotective proteins, such as aldose reductase (AR), Na^+^/Cl^-^-coupled betaine/γ-aminobutyric acid transporter (BGT1), Na^+^-dependent myo-inositol transporter (SMIT), Na^+ ^and Cl^-^-dependent taurine transporter (TauT), UT-A urea transporter, and Hsp70 (reviewed in [18] and [19]). NFAT5-deficient mice suffer severe atrophy of the renal medulla, a naturally hypertonic environment, and impaired lymphocyte function [16,17].

The osmoresponsive function of NFAT5 has been documented in diverse cell types, such as lymphocytes [3,20], embryonic fibroblasts [16,17], kidney cells [16,21], neurons [22,23], and cell lines of different lineages [10]. However, little is known about tonicity thresholds (physiologic or pathologic) at which NFAT5 is activated in specific types of primary cells. In this regard, a transgenic mouse model with an integrated NFAT5-responsive reporter would facilitate the analysis of its transcriptional regulation in primary cells and tissues. An NFAT-luciferase (9xNFAT-Luc) transgenic mouse carrying 9 copies of an NFAT site (5'-TGGAAAATT-3') positioned 5' to the minimal promoter of the α-myosin heavy chain gene was developed by Wilkins et al., who studied the role of the calcineurin-NFATc pathway in cardiac hypertrophy [24]. As described in the original article, luciferase activity was detectable in most organs and was highest in the brain, kidney and heart, indicating that the reporter was functional in different types of tissues. Since the NFAT site used in the reporter construct almost coincided with an optimal binding site for NFAT5 (5'-TGGAAAAAT-3'), we wondered whether it could be activated by this factor.

In this work we show that the 9xNFAT-Luc reporter is activated by NFAT5 in response to hypertonicity in transgenic primary T lymphocytes, macrophages and mouse embryo fibroblasts (MEF), and by NFATc proteins in response to calcineurin activation. Activation of NFAT5 in lymphocytes was detected in response to hypertonicity levels in the range measured in plasma in patients and animal models with osmoregulatory disorders. Activation of NFAT5 transcriptional activity by hypertonicity was substantially downregulated by the p38 inhibitors SB203580 and SB202190, and by inhibition of PIKK with 25 μM LY294002. The reporter was partially sensitive to the calcineurin inhibitor FK506, the PI3-kinase inhibitor wortmannin (0.5 μM), and the protein kinase A inhibitor H89, but was not inhibited by the ERK inhibitor PD98059. These results, together with others in the literature, suggest that activation of NFAT5 by hypertonicity involves different combinations of signaling pathways in different cell types. Our results indicate that 9xNFAT-Luc mice might constitute a useful tool to study the regulation of both NFAT5 and NFATc proteins and the effect of pharmacological modulators in different types of primary cells.

## Results

### Activation of the 9xNFAT-Luc reporter by NFAT5 or NFATc proteins in a stimulus-specific manner

**Figure 1:**
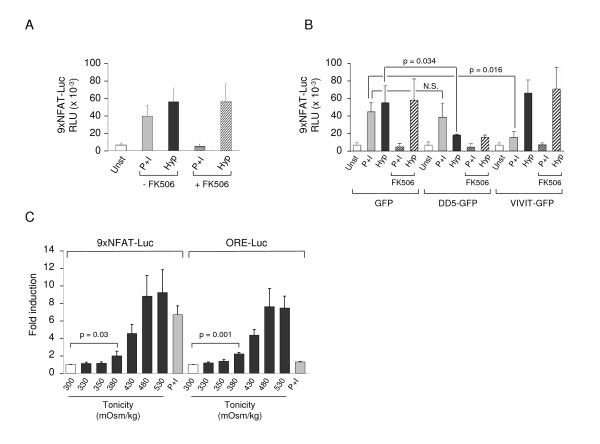
**Activation of the 9xNFAT-luc reporter by NFATc or NFAT5**. **A) **Jurkat T cells transfected with the 9xNFAT-Luc reporter were stimulated with PMA plus ionomycin (P+I) or cultured in hypertonic medium (500 mOsm/kg) without or with FK506 during 24 hours. Luciferase activity is represented as relative light units per second (RLU) after normalization with Renilla and endogenous lactate dehydrogenase. Mean ± S.D of four independent experiments is shown. **B) **9xNFAT-Luc and vectors encoding GFP, the NFAT5-inhibitory dimerization domain (DD5-GFP) or the NFATc-inhibitory peptide VIVIT (VIVIT-GFP) were transfected in Jurkat T cells. Cells were treated during 24 hours with PMA plus ionomycin or hypertonicity (500 mOsm/kg) in the absence or presence of FK506. Mean ± S.D of three independent experiments is shown. N.S.: non statistically significant. **C) **Jurkat T cells transfected with the 9xNFAT-Luc reporter or the ORE-luc reporter were exposed to increasingly hypertonic media in the presence of FK506, or stimulated with PMA plus ionomycin without FK506. Mean ± S.D of three independent experiments is shown.

We observed that the 9xNFAT-Luc reporter was comparably activated by hypertonicity or PMA plus ionomycin (P+I) in the human T lymphocyte cell line Jurkat (Figure 1A). Activation by P+I was suppressed by the calcineurin inhibitor FK506, whereas induction by hypertonicity was not. Hypertonicity-induced activation was downregulated by > 60% in cells transfected with the isolated dimerization domain of NFAT5 (DD5), which inhibits NFAT5 but not NFATc proteins [3], whereas activation by P+I was not significantly inhibited (Figure 1B). The VIVIT peptide, which disrupts the binding of calcineurin to NFATc proteins [25], prevented the activation of the reporter by P+I without affecting its induction by hypertonicity (Figure 1B). The 9xNFAT-Luc reporter was activated by hypertonicity levels between 380 to 530 mOsm/kg, comparably to a widely used NFAT5-dependent reporter driven by the enhancer of the aldose reductase gene [3,26] (Figure 1C). These results indicated that the 9xNFAT-Luc reporter could be activated by distinct types of stimuli: hypertonicity via NFAT5, and PMA plus ionomycin via the calcineurin-dependent NFATc proteins.

**Figure 2:**
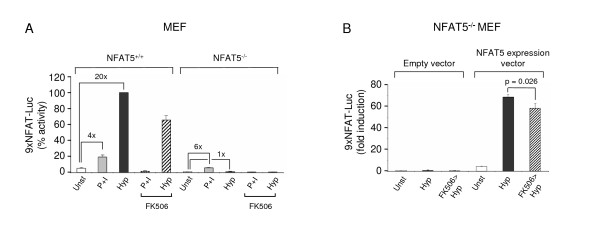
**Unresponsiveness of the 9xNFAT-Luc reporter to hypertonicity in NFAT5-deficient cells**. **A) **NFAT5^+/+ ^and NFAT5^-/- ^MEF were transfected with the 9xNFAT-Luc reporter and stimulated with PMA plus ionomycin or hypertonicity (460 mOsm/kg), with or without FK506, during 24 hours. Mean ± S.D of three independent experiments is shown. **B) **NFAT5^-/- ^MEF were transfected with the 9xNFAT-Luc reporter and an empty vector (pBSK(+)) or an NFAT5 expression vector. Cells were stimulated with PMA plus ionomycin or hypertonicity (460 mOsm/kg), with or without FK506, during 24 hours. Mean ± S.E.M of three independent experiments is shown.

In order to conclusively confirm that the 9xNFAT-Luc reporter was activated by NFAT5 under hypertonic conditions, we bred NFAT5^+/- ^mice [16] into the 9xNFAT-Luc transgenic background to obtain 9xNFAT-Luc^+^/NFAT5^-/- ^mice. We derived mouse embryo fibroblasts (MEF), and also analyzed mature T cells and bone marrow-derived macrophages from several independent NFAT5^-/- ^adult mice. As shown in Figure 2A, hypertonicity activated the 9xNFAT-Luc reporter in NFAT5^+/+ ^MEF, but not in NFAT5^-/- ^cells. Both cell types showed a comparable response to P+I, which was suppressed by FK506. Activation of the reporter by hypertonicity in NFAT5^+/+ ^MEF was partially inhibited (30%) by FK506. Transfection of an NFAT5 expression vector in NFAT5^-/- ^MEF reconstituted their responsiveness to hypertonicity (Figure 2B). Results obtained with 9xNFAT-Luc transgenic T cells derived from NFAT5^+/+ ^and NFAT5^-/- ^mice confirmed that activation of the reporter by hypertonicity was severely impaired in NFAT5^-/- ^cells, whereas activation by P+I was independent of NFAT5 (Table 1). Hypertonicity-induced activation of the 9xNFAT-Luc reporter was variably inhibited by FK506 in T lymphocytes. We also noticed that hypertonicity induced a weak activation of the reporter in NFAT5^-/- ^cells, which was also partially inhibited by FK506. It has been recently shown that NFATc1 can be activated by hypertonicity [27], and thus it is possible that the residual activity induced by hypertonic stimulation in NFAT5^-/- ^T cells could be due to NFATc proteins. Nonetheless, our overall results in primary T lymphocytes, macrophages (see below), MEF and Jurkat T cells showed that activation of the 9xNFAT-Luc reporter by hypertonicity was predominantly attributable to NFAT5, while other factors made a much lesser contribution to this stimulus. On the other hand, we did not observe significant contribution of NFAT5 to the activation of the 9xNFAT-Luc reporter by P+I.

**Table 1:** Impaired activation of endogenous 9xNFAT-Luc transgenic reporter by hypertonicity in NFAT5-deficient lymphocytes.

		9xNFAT-Luc reporter activity (RLU)					
		
		Experiment # 1		Experiment # 2		Experiment # 2	
				
		NFAT5 +/+	NFAT5 -/-	NFAT5 +/+	NFAT5 -/-	NFAT5 +/+	NFAT5 -/-
*8-hour stimulation*							
						
300 mOsm/kg (isotonic)	- FK506	19	25	45	19	16	16
	+ FK506	11	13	18	16	7	8
430 mOsm/kg (hypertonic)	- FK506	1026	117	825	191	387	27
	+ FK506	720	83	1316	13	579	24
480 mOsm/kg (hypertonic)	- FK506	12616	540	5677	42	10901	273
	+ FK506	19543	435	2894	51	7403	102
PMA+ ionomycin (isotonic)	- FK506	2445	10087	2080	2494	1726	6364
	+ FK506	24	19	34	31	37	158
							
*24-hour stimulation*							
						
300 mOsm/kg (isotonic)	- FK506	21	21	21	20	19	33
	+ FK506	10	15	14	15	12	10
430 mOsm/kg (hypertonic)	- FK506	1351	188	297	239	348	102
	+ FK506	550	122	254	129	388	73
480 mOsm/kg (hypertonic)	- FK506	6930	359	1742	224	7267	245
	+ FK506	1507	232	1111	129	3977	129
PMA+ ionomycin (isotonic)	- FK506	1100	2516	791	825	418	1775
	+ FK506	15	17	16	8	30	30

Luciferase activity was measured in proliferating transgenic 9xNFAT-Luc T cells derived from littermate NFAT5^+/+ ^and NFAT5^-/- ^mice after stimulation with hypertonic medium or PMA plus ionomycin, in the absence or presence of 100 nM FK506. Luciferase activity is represented as relative light units per second (RLU) after normalization with endogenous lactate dehydrogenase.

### Hypertonicity threshold for NFAT5 activation

Studies on hypertonic stress responses in different types of mammalian cells usually utilize hypertonicity levels of 500 mOsm/kg or higher, although it is poorly understood where cells might be exposed to such elevated hypertonicity levels besides the renal medulla. Physiologic osmolality values in plasma, brain and lung in mice are close to 300 mOsm/kg, and between 330–340 mOsm/kg in thymus, spleen and liver [17]. In humans, normal plasma osmolality is ~290 mOsm/kg, but can rise to the range of 380 to 430 mOsm/kg in cases of severe hypernatremia [28], salt poisoning in infants [29], adipsic disorders with impairment of thirst perception [30-32], and renal pathologies [33,34]. Constitutively elevated plasma tonicity (~400 mOsm/kg) has been reported in mice deficient in V2 vasopressin receptor [35], and in mice with congenital progressive hydronephrosis caused by a mutation in aquaporin-2 [36]. In aquaporin-1-deficient mice, plasma tonicity can reach 517 mOsm/kg after 36 hours of water deprivation, despite which these can survive if water is administered to them again [37]. Elevation of the tonicity in plasma would expose different tissues to hypertonic stress and might activate NFAT5, as shown in rats where an acute rise of plasma osmolality to 420 mOsm/kg triggered a rapid increase in expression and nuclear accumulation of NFAT5 in neurons [22].

**Figure 3:**
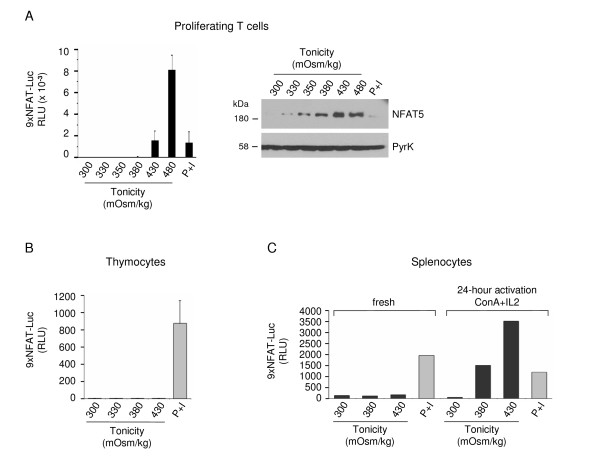
**Activation of the transgenic 9xNFAT-Luc reporter in fresh and mitogen-activated lymphocytes**. **A) **Proliferating transgenic 9xNFAT-Luc T cells were exposed to increasingly hypertonic media in the presence of FK506, or stimulated with PMA plus ionomycin during 24 hours. Left panel: luciferase activity (RLU) shown is the mean ± S.E.M of five independent experiments. Right panel: NFAT5 and pyruvate kinase (protein loading control) were detected by Western blotting. One representative experiment is shown out of three performed independently. **B) **Luciferase activity in 9xNFAT-Luc transgenic thymocytes exposed to increasingly hypertonic media in the presence of FK506, or stimulated with PMA plus ionomycin during 24 hours. Mean ± S.E.M of three independent experiments is shown. **C) **Luciferase activity in 9xNFAT-Luc transgenic splenocytes stimulated during 24 hours with hypertonicity or PMA plus ionomycin, either immediately after their purification, or after a 24-hour preactivation with concanavalin A plus IL2. One representative experiment is shown out of three performed independently.

**Figure 4:**
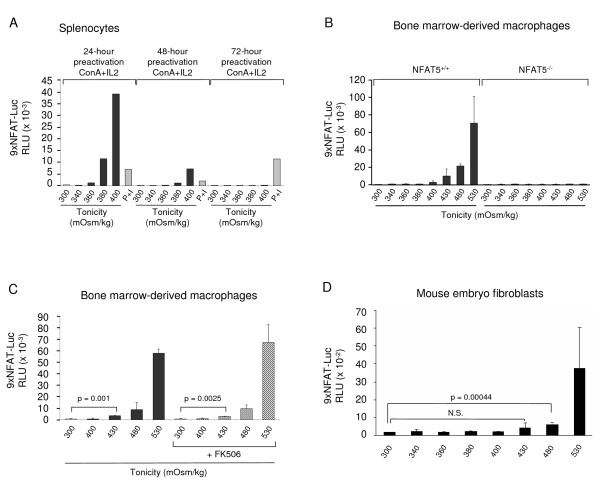
**Hypertonicity threshold required to activate the 9xNFAT-Luc reporter in different cell types**. **A) **Luciferase activity in 9xNFAT-Luc transgenic splenocytes treated during 24 hours with hypertonicity or PMA plus ionomycin, after having been preactivated with concanavalin A plus IL2 during 24 hours, 48 hours or 72 hours. One representative experiment is shown out of three performed independently (see Table 2). **B) **Luciferase activity in 9xNFAT-Luc transgenic bone marrow-derived macrophages from NFAT5^+/+ ^and NFAT5^-/- ^mice exposed to increasing hypertonicity levels during 24 hours. Mean ± S.D of two independent experiments is shown. **C) **Luciferase activity in 9xNFAT-Luc transgenic bone marrow-derived macrophages exposed to increasing hypertonicity levels during 24 hours, in the absence or presence of FK506 (100 nM). Mean ± S.D of three independent experiments is shown. **D) **Luciferase activity in 9xNFAT-Luc transgenic mouse embryo fibroblasts exposed to increasing hypertonicity levels during 24 hours. Mean ± S.D of three independent experiments is shown. N.S.: non statistically significant.

Titration of the responsiveness of the 9xNFAT-Luc reporter to hypertonicity in the T cell line Jurkat showed that the reporter was significantly activated by hypertonicity levels of ≥ 380 mOsm/kg (Figure 1C). Proliferating T cells derived from splenocytes stimulated during 3 days with the mitogen concanavalin A (ConA) plus IL2 showed calcineurin-independent activation of the reporter at 430 mOsm/kg (Figure 3A). In the same type of cells, induction of NFAT5 expression was detected at lower tonicity values (330 mOsm/kg) than the activation of the reporter. In these experiments, stimulation with hypertonicity was done in the presence of the calcineurin inhibitor FK506 to prevent any potential contribution of NFATc proteins. When we measured the activity of the reporter in freshly isolated thymocytes and splenocytes, we detected its activation only in response to P+I, but not with hypertonicity (Figures 3B and 3C). These results suggested that optimal NFAT5-mediated activation of the transgenic reporter in lymphocytes depended on their activation state. In agreement with this, we found that a 24-hour stimulation of T cells with mitogens conferred them the ability to respond to hypertonicity levels of 380 mOsm/kg (Figure 3C). Next, we analyzed whether the responsiveness of the reporter varied at different time points after T cells had been stimulated with mitogens. As shown in Figure 4A and Table 2, the response to hypertonicity was strongest when cells had been stimulated with mitogens for one day. In these cells, activation of the 9xNFAT-Luc reporter was robustly induced at 380 and 400 mOsm/kg, and was even detectable at 360 mOsm/kg in some of the experiments. The intensity of the response became weaker in T cells that had been cultured during 48 hours or longer (Figure 4A and Table 2). We also found that lymphocytes showed a lower hypertonicity threshold for NFAT5 activation than bone marrow-derived macrophages and MEF. As shown in Figure 4B, NFAT5-dependent activation of the 9xNFAT-Luc reporter in macrophages was observed at 430 mOsm/kg. It was noticeable that hypertonicity-induced activation of this reporter was insensitive to FK506 in macrophages. Activation of the reporter in MEF required a higher hypertonicity threshold (480 mOsm/kg) (Figure 4C).

**Table 2:** Hypertonicity threshold required to activate the endogenous 9xNFAT-Luc reporter in transgenic lymphocytes.

		9xNFAT-Luc reporter activity (RLU)			
		
Preactivation with ConA + IL2	Stimulus	Experiment # 1	Experiment # 2	Experiment # 3	Experiment # 4
*24 hours*	300 mOsm/kg (isotonic)	398	446	761	662
	340 mOsm/kg	295	673	1628	690
	360 mOsm/kg	1254	933	1648	944
	380 mOsm/kg	11468	1622	6060	2353
	400 mOsm/kg	38768	22808	11061	6494
	PMA + ionomycin (isotonic)	7039	20428	12081	6647
					
*48 hours*	300 mOsm/kg (isotonic)	257	351	329	210
	340 mOsm/kg	159	397	441	311
	360 mOsm/kg	205	327	567	226
	380 mOsm/kg	1118	1121	1204	1258
	400 mOsm/kg	7105	7997	1891	3291
	PMA + ionomycin (isotonic)	2079	19702	1803	3638
					
*72 hours*	300 mOsm/kg (isotonic)	155	356	113	146
	340 mOsm/kg	136	357	131	214
	360 mOsm/kg	128	342	196	99
	380 mOsm/kg	188	379	260	148
	400 mOsm/kg	288	467	458	288
	PMA + ionomycin (isotonic)	11359	22321	2389	1511

Transgenic 9xNFAT-Luc splenocytes were activated with concanavalin A plus IL2 during 24 to 72 hours in isotonic medium, and then exposed to hypertonicity, or stimulated with PMA plus ionomycin for 24 hours. Luciferase activity is represented as relative light units per second (RLU) after normalization with endogenous lactate dehydrogenase.

### Sensitivity of NFAT5 transcriptional activity to pharmacological inhibitors

**Figure 5:**
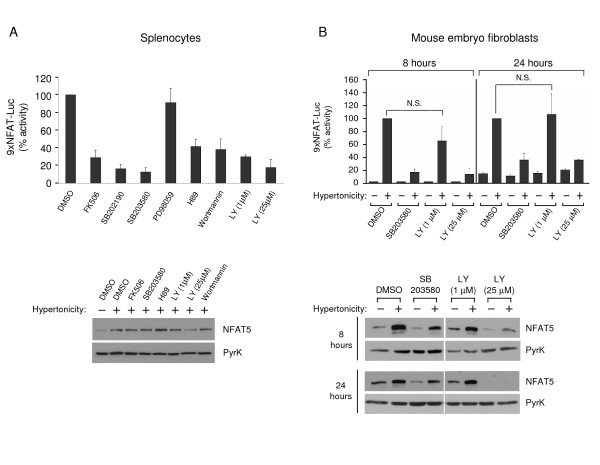
**Sensitivity of NFAT5 transcriptional activity and expression to pharmacological inhibitors**. **A) **Transgenic 9xNFAT-Luc splenocytes preactivated during 24 hours with concanavalin plus IL2 were treated with hypertonicity (400 mOsm/kg, 24 hours) in the absence or presence of FK506 (100 nM), SB203580 or SB202190 (both at 10 μM), PD98059 (10 μM), H89 (2 μM), wortmannin (0.5 μM), or LY294002 (LY). Luciferase activity in the upper panel (mean ± S.D of three independent experiments) is shown as percentage of the activity in the absence of inhibitors (100%). Western blotting in the lower panelshows NFAT5 and pyruvate kinase expression. **B) **Transgenic MEF were stimulated for 8 or 24 hours with hypertonicity (460 mOsm/kg) in the absence or presence of SB203580 (10 μM) or LY294002 (LY). Luciferase activity is shown is the upper panel (mean ± S.E.M of three independent experiments). NFAT5 and pyruvate kinase expression are shown in the lower panel.

NFAT5 is fundamental in the adaptation of mammalian cells to osmotic stress, and the regulation of its transcriptional function by signaling pathways in primary, non-transformed cells, has not been fully elucidated. We analyzed the transcriptional response to hypertonicity in T cells treated with inhibitors of signaling pathways that had been reported to regulate NFAT5 in other cell types. As shown in Figure 5A, activation of the 9xNFAT-Luc reporter was downregulated by the p38 inhibitors SB203580 and SB202190 (both at 10 μM), and by inhibition of PIKK with 25 μM LY294002. The reporter was insensitive to the ERK inhibitor PD98059 (10 μM), and partially inhibited by the calcineurin inhibitor FK506 (100 nM), the PI3-kinase inhibitors wortmannin (0.5 μM) and LY294002 (1 μM), and the protein kinase A inhibitor H89 (2 μM). In the same type of experiment, 25 μM LY294002 also caused a mild inhibition of NFAT5 expression (Figure 5A). The JNK inhibitor SP600125 (10 μM) yielded inconsistent results, and we only observed a 30% inhibition of the reporter in one of three independent experiments (not shown).

Experiments in MEF showed that activation of the 9xNFAT-Luc reporter was also prevented by SB203580 and LY294002 (Figure 5B). In these cells, SB203580 partially reduced the accumulation of NFAT5 in response to hypertonicity, while 25 μM LY294002 caused a substantial inhibition of NFAT5 expression. These results revealed that LY294002-sensitive kinases regulated both the transcriptional activation of NFAT5 and its expression in response to hypertonicity in different cell types.

## Discussion

We show that the 9xNFAT-Luc reporter integrated in transgenic cells is activated by endogenous NFAT5 in response to hypertonicity and by NFATc proteins in response to calcineurin activation. A previous report had shown that a chimeric reporter with NFAT5 sites inserted into the minimal IL2 promoter could be activated by NFAT5 in response to hypertonicity as well as PMA plus ionomycin in Jurkat T cells [38], suggesting that this factor might regulate certain promoters in response to non-hypertonic stimuli. Our results in primary mouse lymphocytes, macrophages, MEF and Jurkat cells show a remarkable selectivity in the activation of the 9xNFAT-Luc reporter by NFAT5 in response to hypertonicity, and by NFATc proteins in response to PMA plus ionomycin. These experiments indicate that the specific recruitment of either NFATc or NFAT5 to DNA sites to which both factors can bind may be determined by the type of stimulus. This finding is in line with work by the Goldfeld laboratory, who showed that some sites in the TNFα promoter and the LTR of HIV could recruit both NFAT5 and NFATc [39,40], and that occupancy of the site by either type of transcription factor depended on the stimulus. Hypertonicity-mediated activation of the 9xNFAT-Luc reporter was variably inhibited by FK506 in primary T cells and MEF, although not in macrophages and Jurkat cells, suggesting that calcineurin can modulate the hypertonic stress response in some cell types. This observation is consistent with a recent report showing that calcineurin was a positive regulator of both NFATc1 and NFAT5 in the activation of the aquaporin 2 promoter by hypertonicity in murine collecting duct cells [27]. Altogether, results from previous work [3,27,38], and ours here indicate that calcineurin might enhance the activation of NFAT5 by hypertonicity in some cell types, although it is not generally essential for its function, in contrast to the strict requirement of this phosphatase in the activation of NFATc proteins.

The variable dependence of NFAT5 transcriptional activity on calcineurin in different cell types would need to be considered when using the 9xNFAT-Luc mice to study the activation of NFAT5 and NFATc proteins *in vivo *or in primary cultures from different organs. The use of calcineurin inhibitors in cell culture experiments, and parallel analysis of NFAT5-deficient cells, as we have shown here, would ensure that the measured reporter activity is derived from NFAT5 rather from NFATc. *In vivo*, however, calcineurin inhibitors might complicate the interpretation of results due to side effects such as nephrotoxicity, which might affect sodium and water homeostasis in the organism [41] and indirectly perturb the regulation of NFAT5 and NFATc proteins. Crosses between 9xNFAT-Luc mice and tissue-specific conditional knockout for calcineurin or NFAT proteins might prove useful to study these factors *in vivo*.

Our experiments, together with independent results from the literature, revealed some heterogeneity in the sensitivity of NFAT5 transcriptional activity to pharmacological inhibitors. Hypertonicity-induced activation of NFAT5 in T lymphocytes was substantially downregulated by the PI3-kinase and PIKK inhibitors wortmannin and LY294002. This result agreed with previous reports using Jurkat T cells and HEK293 cells [10,11]. We also found that inhibition of PIKK impaired the upregulation of NFAT5 expression by hypertonicity in non-transformed lymphocytes and MEF. ATM and other PIKK regulate the nuclear translocation of NFAT5 [42], but their involvement in upregulating NFAT5 expression had not been previously documented. These observations indicate that PIKK regulate different layers of NFAT5 function.

With regard to other pathways, we found that activation of NFAT5 was inhibited by two independent p38 inhibitors, SB203580 and SB202190. Reports indicate that p38 might not be a universal regulator of NFAT5, since Kultz et al., showed that a dominant negative construct of MKK3 failed to inhibit the NFAT5-dependent reporter ORE-Luc in PAP-HT25 rabbit renal medullary cells [12], whereas Ko et al., showed that both a dominant negative p38 construct and SB203580 effectively inhibited the same reporter in NIH3T3 and MEF [7]. Tsai et al., found that a dominant negative p38 also inhibited the NFAT5-regulated TauT and Hsp70 promoters in nucleus pulposus cells [9]. The same report also showed that a dominant negative ERK construct and the ERK inhibitor PD98059 partially inhibited NFAT5, whereas we found that activation of NFAT5 in primary mouse T cells was insensitive to PD98059. H89 had been reported to partially inhibit NFAT5 transcriptional activity in the HepG2 hepatocellular carcinoma cell line [8], and we obtained similar results in T cells. The JNK inhibitor SP600125 did not impair the activation of the 9xNFAT-Luc reporter in T cells (not shown), in agreement with previous reports showing that neither SP600125 [9] nor inhibition of JNK with a dominant negative SEK1 construct [12] inhibited NFAT5 in other cell types. We interpret our results with inhibitors cautiously, as some might inhibit more than one single kinase (see [43,44], and [45] for a recent update). Nonetheless, our profiling of the sensitivity of NFAT5 to pharmacological inhibitors in T cells, together with previous reports, supports the notion that activation of NFAT5 by hypertonicity involves distinct combinations of regulators in different cell types. Unravelling the identity of kinases and properties of NFAT5-regulatory pathways in specific cell types would help to understand the relevance of osmotic stress responses in mammals.

While it is clear that the osmoprotective function of NFAT5 is critical in the renal medulla, a physiologically hypertonic environment [16], many other cell types can also express and activate this factor when exposed to hypertonic stress. However, its activation threshold under physiological or pathological tonicity conditions in different cell types is largely unknown. Our experiments show that activated T lymphocytes are sensitive to moderate increases in extracellular tonicity, and could induce NFAT5 expression at relatively low levels of hypertonicity (330 mOsm/kg), and detectable transcriptional activity of this factor at 360–380 mOsm/kg, which increased sharply at 400 mOsm/kg. Tonicity levels in the 360–400 mOsm/kg range have been recorded in the plasma of patients with osmoregulatory disorders and in aquaporin and vasopressin receptor-deficient mice [28-37]. The hypertonicity level required to stimulate the 9xNFAT-Luc reporter in lymphocytes varied with their activation state. The reporter did not respond to hypertonicity in resting lymphocytes, showed a robust response to 380 and 400 mOsm/kg in T cells that had been exposed to mitogens for 24 hours, and became less responsive in cells cultured during 48 to 72 hours. T cell activation by mitogens or antigen receptor causes a dramatic increase in biogenesis, cell growth and gene expression during the first 24–48 hours, that precedes the entry of the lymphocyte in a series of cell division rounds [46,47]. That T cells display a greater sensitivity to hypertonicity and a stronger NFAT5 transcriptional response in the first stages of their activation is likely relevant to ensure their function under potentially harmful osmotic stress conditions. In this regard, exposure of fresh NFAT5-deficient T cells to 370 mOsm/kg during the first 3 days of antigen receptor-induced activation reduced their proliferation rate by more than 60%, whereas normal lymphocytes were not affected [17]. Altogether, these observations indicate that pathologic elevations of the extracellular tonicity rapidly activate the expression and transcriptional activation of NFAT5 in lymphocytes to ensure an appropriate osmoprotective response.

Activation of the reporter in macrophages and MEF required higher tonicity levels than in lymphocytes. It is intriguing that diverse cell types appear to require different hypertonicity levels to activate NFAT5-mediated transcriptional responses. While lymphocytes, as shown here and by Go et al. [17], and possibly neurons [22] and macrophages can respond to hypertonicity levels that may occur in certain pathologic conditions, other cells such as fibroblasts require a hypertonicity threshold that is unlikely to be found out of the renal medulla. Investigating the gene expression programs regulated by increasing levels of hypertonicity in different cell types might provide clues about the biological relevance of this response.

## Conclusion

Our study indicates that the 9xNFAT-Luc reporter can be selectively activated by NFAT5 or NFATc proteins in a stimulus-specific manner. Transgenic 9xNFAT-Luc mice might be used to analyze not only NFATc proteins, but also the transcriptional activation of NFAT5 by hypertonicity and its regulation by signaling pathways in primary cells and tissues. It was known that NFAT5 is fundamental to sustain cell function and viability in the renal medulla, a naturally hypertonic environment. Here we show that the expression and transcriptional activation of NFAT5 in lymphocytes are remarkably sensitive to pathologic disturbances of the extracellular tonicity. Similar studies using this transgenic mouse model combined with other approaches should help to elucidate the role of NFAT5 in response to physiopathological tonicity changes in different types of mammalian cells.

## Methods

### Reagents

Phorbol 12-myristate 13-acetate (PMA), the calcium ionophore ionomycin, FK506, and the protein kinase inhibitors H89, LY294002, PD98059, SB202190, SB203580, SP600125 and wortmannin were purchased from Calbiochem (Darmstadt, Germany).

### Cell culture

The human T cell line Jurkat (Clone E6-1, American Type Culture Collection, #TIB 152) was kindly provided by Jeremy Luban (Columbia University College of Physicians and Surgeons, New York, NY) and maintained in Dulbecco's modified Eagle's Medium (DMEM) supplemented with 10% heat-inactivated fetal bovine serum, 2 mM L-glutamine, 1 mM sodium pyruvate, and 50 μM β-mercaptoethanol (Gibco, Pasley, UK). Mouse embryonic fibroblasts (MEF), bone marrow-derived macrophages (BMDM), and mouse T lymphocytes were cultured in the above medium plus 100 μM non-essential amino acids and penicillin-streptomycin (Gibco).

### Mouse embryonic fibroblasts, macrophages, and lymphocytes

9xNFAT-Luc mice (line 15.1) [24] in FVB background, and NFAT5^+/- ^mice [16] in 129Sv background were bred and maintained under specific pathogen-free conditions, and handled according to institutional guidelines (PRBB Animal Care and Use Committee). MEF were prepared from 13.5-day embryos using the NIH3T3 protocol to obtain spontaneously immortalized cells [16]. Bone marrow-derived macrophages (BMDM) were obtained by culturing femur and tibia marrow cell suspensions in L-929 cell-conditioned medium as previously described [48]. Mouse L-929 cells were kindly provided by Antonio Celada (Barcelona Institute for Biomedical Research, Barcelona, Spain). Briefly, bone marrow cells were cultured (37°C, humidified 5% CO_2 _atmosphere) in plastic tissue culture dishes (150 mm) in 40 ml of DMEM containing 10% FBS and 30% L-929 cell-conditioned medium as a source of M-CSF. Penicillin/Streptomycin were added. After 7 days of culture, macrophages were obtained as a homogenous population of adherent cells (>95% CD11b^+^). No differences were observed in the expansion and differentiation of macrophages derived from NFAT5^+/+ ^or NFAT5^-/- ^mice (not shown). Splenocytes and thymocytes were isolated from 8–12 weeks old mice. Proliferating T cells were obtained by activating splenocytes with 2.5 μg/ml concanavalin A (Sigma, Steinheim, Germany) plus 25 ng/ml of recombinant human IL2 (Proleukin, Chiron, Amsterdam, The Netherlands) for three days. T lymphocytes (>95% CD3^+^) were cultured at 1 × 10^6 ^cells/ml in fresh medium supplemented with IL2 for an additional 24 hours. Cells growing in IL2-supplemented isotonic medium (300 mOsm/kg) were treated with 10 nM PMA plus 0.3 μM ionomycin, or hypertonicity, by adding NaCl. Osmolarity of the culture medium was measured in a Fiske One-Ten Osmometer (Fiske Associates. Norwood, MA, USA). Over an isotonic baseline of 300 mOsm/kg, addition of 30 mM NaCl raised the osmolality to 360 mOsm/kg, 40 mM NaCl to 380 mOsm/kg, 50 mM NaCl to 400 mOsm/kg, and 90 mM NaCl to 480 mOsm/kg.

### DNA constructs

The luciferase reporters 9xNFAT-Luc [24] and ORE-luc have been described [3]. Expression vectors for NFAT5 (Myc-NFAT5-GFP) [2], NFAT5 dimerization domain (DD5-GFP) [3], and the NFATc inhibitory peptide VIVIT (VIVIT-GFP) [25] were described. pEGFP-N1 (Clontech, Palo Alto, CA, USA), pTK-Renilla (Promega, Madison, WI, USA), and pBlueScript SK+ (pBSK+) (Stratagene, La Jolla, CA, USA) are available commercially.

### Transfections and reporter assays

Jurkat T cells were transfected by electroporation (Bio-Rad Gene Pulser. Bio-Rad, Hemel Hampstead, UK) [49], with luciferase reporter plasmids (0.3 μg/10^6 ^cells) and TK-Renilla (0.1 μg/10^6 ^cells), together with either pEGFP-N1 (1 μg/10^6 ^cells), VIVIT-GFP (2 μg/10^6 ^cells), or DD5-GFP (2 μg/10^6 ^cells). MEF were transfected by the calcium-phosphate method in 10-cm plates with luciferase reporter (1 μg/plate) plus TK-Renilla (1 μg/plate), and either pBSK+ (22 μg/plate) or NFAT5 expression vector (22 μg/plate). Transfected cells were stimulated in isotonic medium (300 mOsm/kg) with either 20 nM PMA plus 1 μM ionomycin or subjected to hypertonic conditions as indicated in figure legends. FK506 was used at 100 nM. Luciferase and Renilla were measured with the Dual-luciferase reporter system (Promega) with a Berthold FB12 luminometer (Berthold, Pforzheim, Germany). When reporters were transfected in cell lines, luciferase activity was normalized to Renilla and endogenous lactate dehydrogenase (LDH), which was proportional to the number of viable cells [49]. Luciferase activity in transgenic cells was normalized to endogenous LDH in the same lysate, measured with the CytoTox 96 Non-Radioactive Cytotoxicity Assay (Promega).

### Western blot

Cell lysates, Western blotting, and enhanced chemiluminescent detection (Supersignal West Pico Chemiluminescent Substrate, Pierce, Rockford, IL, USA) were done as described [3]. PVDF membranes were probed with anti-NFAT5 (catalog number: PA1-023, Affinity Bioreagents; Golden CO, USA). Anti-Pyruvate kinase (AB1235; Chemicon, Hampshire, UK) was used as a protein loading control.

## Authors' contributions

BM performed the experiments and participated in the design and writing of the manuscript. JM set up the culture of bone marrow-derived macrophages and did their phenotypical analysis. JDM provided the 9xNFAT-Luc transgenic mice and critically reviewed the manuscript. CLR provided NFAT5-deficient mice, isolated the MEF used in this study, and contributed to the design and drafting of the manuscript. JA designed the study, supervised the experiments and wrote the manuscript. All authors read and approved the final manuscript.
